# Differential Macrophage Activation Alters the Expression Profile of NTPDase and Ecto-5′-Nucleotidase

**DOI:** 10.1371/journal.pone.0031205

**Published:** 2012-02-13

**Authors:** Rafael Fernandes Zanin, Elizandra Braganhol, Letícia Scussel Bergamin, Luís Felipe Ingrassia Campesato, Alfeu Zanotto Filho, José Cláudio Fonseca Moreira, Fernanda Bueno Morrone, Jean Sévigny, Maria Rosa Chitolina Schetinger, Angela Terezinha de Souza Wyse, Ana Maria Oliveira Battastini

**Affiliations:** 1 Departamento de Bioquímica, Instituto de Ciências Básicas da Saúde, Universidade Federal do Rio Grande do Sul, Porto Alegre, Rio Grande do Sul, Brasil; 2 Faculdade de Farmácia, Pontifícia Universidade Católica do Rio Grande do Sul, Porto Alegre, Rio Grande do Sul, Brasil; 3 Centre de Recherche en Rheumatologie et Immunologie, Centre Hospitalier Universitaire de Québec (Pavillon CHUL); 4 Departamento de Química, Centro de Ciências Naturais e Exatas, Universidade Federal de Santa Maria, Santa Maria, Rio Grande do Sul, Brasil; 5 Département de microbiologie-infectiologie et d'immunologie, Faculté de médecine, Université Laval, Québec, Québec, Canada; University of Colorado Denver, United States of America

## Abstract

Macrophages are key elements in the inflammatory process, whereas depending on the micro-environmental stimulation they exhibit a pro-inflammatory (classical/M1) or an anti-inflammatory/reparatory (alternative/M2) phenotype. Extracellular ATP can act as a danger signal whereas adenosine generally serves as a negative feedback mechanism to limit inflammation. The local increase in nucleotides communication is controlled by ectonucleotidases, such as members of the ectonucleoside triphosphate diphosphohydrolase (E-NTPDase) family and ecto-5′-nucleotidase/CD73 (ecto-5′-NT). In the present work we evaluated the presence of these enzymes in resident mice M1 (macrophages stimulated with LPS), and M2 (macrophages stimulated with IL-4) macrophages. Macrophages were collected by a lavage of the mice (6–8 weeks) peritoneal cavity and treated for 24 h with IL-4 (10 ng/mL) or LPS (10 ng/mL). Nitrite concentrations were measured using the Greiss reaction. Supernatants were harvested to determine cytokines and the ATPase, ADPase and AMPase activities were determined by the malachite green method and HPLC analysis. The expression of selected surface proteins was evaluated by flow cytometry. The results reveal that M1 macrophages presented a decreased ATP and AMP hydrolysis in agreement with a decrease in NTPDase1, -3 and ecto-5′-nucleotidase expression compared to M2. In contrast, M2 macrophages showed a higher ATP and AMP hydrolysis and increased NTPDase1, -3 and ecto-5′-nucleotidase expression compared to M1 macrophages. Therefore, macrophages of the M1 phenotype lead to an accumulation of ATP while macrophages of the M2 phenotype may rapidly convert ATP to adenosine. The results also showed that P1 and P2 purinoreceptors present the same mRNA profile in both phenotypes. In addition, M2 macrophages, which have a higher ATPase activity, were less sensitive to cell death. In conclusion, these changes in ectoenzyme activities might allow macrophages to adjust the outcome of the extracellular purinergic cascade in order to fine-tune their functions during the inflammatory set.

## Introduction

Macrophages play key functions in the inflammatory process and are characterized by a marked phenotypic heterogeneity depending on their micro-environmental stimulation [1]–[2]. These cells exhibit diverse biochemical properties that influence pathobiology with classical/M1 and alternative/M2 polarization representing phenotypic extremes [3]. Classical activation is induced by microbial agents and/or T helper cell type 1 (Th1) cytokines and interferon-γ (IFN-γ), being associated with the production of large amounts of nitric oxide (NO) and pro-inflammatory cytokines (IL-1β, IL-6, IL-12 and TNF-α), which are involved in cytotoxicity and microbial killing [4]–[5]. In contrast, alternative activation is induced by Th2 cytokines (IL-4 and/or IL-13), and is characterized by anti-inflammatory and tissue repair properties [3]. IL-4 stimulates the production of anti-inflammatory cytokines such as IL-10 and IL-1R antagonist [6] and inhibits the production of pro-inflammatory cytokines [7]–[8], thus reducing inflammation. Moreover, alternative activated macrophages are characterized by an increase in the extracellular matrix remodeling associated with the expression of matrix proteins, such as fibronectin, βIGH3, fibrogenesis, and a high expression of arginase, which is related to repair properties [9]–[12].

Macrophages can also respond to endogenous stimuli that are rapidly generated following injury [1]. Nucleotides and nucleosides are currently considered as true inflammatory mediators [13]–[16]. The concentration of nucleotides/nucleosides in the extracellular space is maintained at low levels by ectonucleotidases and adenosine transporters, but accumulation of these molecules may occur in some situations such as mechanical stress, cell injury and inflammation. Extracellular ATP can act as a danger signal molecule initiating an innate immune response. For example, this nucleotide induces the macrophages to release a repertory of pro-inflammatory cytokines such as IL-1β and IL-6 and superoxide [17]–[21]. In contrast, the ATP breakdown product adenosine serves as a negative feedback mechanism, limiting inflammation by suppressing the actions of immune cells [17], [22].

Biological effects of extracellular nucleotides (e.g. ATP, UTP) and nucleosides (adenosine) are evoked by activating transmembrane receptors of the P2 and P1 family [23], respectively, which are expressed by all cells including monocytes/macrophages. Primary monocytes express ion-channel P2X1,4,5,7 and G-protein-coupled P2Y_1,2,4,6,11–13_ receptor mRNAs, while macrophages express the same receptor subtypes except P2Y_13_ [24]–[26]. P2Y_1_, P2Y_2_, P2X4 and P2X7 receptors have been detected in macrophages at protein level [24]–[26]. All four (A_1_, A_2A_, A_2B_ and A_3_) adenosine receptors are expressed by both monocytes and macrophages [17], [22].

The concentrations of ATP, UTP and adenosine are controlled by ectonucleotidases, such as members of the ectonucleoside triphosphate diphosphohydrolase (E-NTPDase) family and ecto-5′-nucleotidase/CD73 (ecto-5′-NT) [27]–[28]. NTPDases efficiently hydrolyze nucleoside 5′-triphosphates and diphosphates (e.g. ATP, UTP, ADP, UDP) to the respective nucleoside-5′-monophosphate derivative, with considerable difference in their preference for individual type of nucleotide. In mammals, eight E-NTPDase members named NTPDase1–8 have been cloned and characterized [27]. Four of these NTPDases are typical cell surface-located enzymes with an extracellular catalytic site (NTPDase1, -2, -3, -8). NTPDase1/CD39 hydrolyzes ATP and ADP with comparable rates producing the rapid formation of AMP as a final product. NTPDase2 exhibits high preference for ATP and therefore, could favor extracellular ADP accumulation [29]. NTPDase3 and -8 reveal an intermediary rate preference for ATP over ADP. AMP produced by NTPDase activity is further hydrolyzed to adenosine by ecto-5′-NT [27]–[28], [30]. In the vasculature and in blood cells, NTPDase1/CD39 and ecto-5′-NT are expressed by leukocytes [31]–[32], endothelial cells [33] and platelets [32], [34] being involved in the inhibition of platelet recruitment and thrombus formation [35], leukocyte migration [36] and immunosuppressive functions [37].

Hence, considering the importance of the macrophage spectrum activation and the purinergic signaling in the innate immune responses, we hypothesized that ectonucleotidases, by modulating the P2/P1 receptor activation, might participate in macrophage differentiation and/or be involved in the functions played by different macrophages. Accordingly, in the present work we show that NTPDases and ecto-5′-NT are differentially expressed in resident, classical and alternative activated macrophages. The participation of the ectonucleotidase pathway along the differential macrophage activation process is further discussed, providing new insights on the role of purinergic signaling in the phenotypic modulation of immune cells.

## Materials and Methods

### Animals and Reagents

Swiss male mice, 6–8 weeks-old, were maintained under a standard dark–light cycle (lights on between 7:00 a.m. and 7:00 p.m.) at room-controlled temperature (22±2°C). The mice had free access to standard laboratory chow and water. The animal handling and experiments were performed in accordance with the international guidelines in compliance with the Federation of Brazilian Societies for Experimental Biology and approved by the Ethics Committee for the Use of Animals of Pontifícia Universidade Católica do Rio Grande do Sul (CEUA-PUCRS) under protocol ID CEUA 09/0005. All chemicals were purchased from Sigma Chemical Co. (St. Louis, MO, USA).

### Macrophages activation

Peritoneal macrophages were collected by lavage of the peritoneal cavity with 5 mL of sterile RPMI-1640 medium without fetal bovine serum (FBS). The cells were washed twice with sterile Phosphate Buffered Saline (PBS) and suspended in RPMI without FBS. The obtained cells were transferred to 6, 24 or 48 multi-well plates and allowed to attach for 30 min. Unattached cells were washed out with RPMI without FBS. The attached cells, mainly peritoneal macrophages, were used for the experiments thereafter. Macrophages were evaluated by microscopic examination of the culture wells after May-Grunwald and Giemsa stains, indicating macrophage purity higher than 80%, which was confirmed using the CD11b Ab.

The obtained macrophages were stimulated for 24 h in complete medium (RPMI plus 10% FBS) with LPS (100 ng/mL) or IL-4 (10 ng/mL) (Sigma) for the generation of classically or alternative macrophage activation, respectively. Resident macrophages were maintained in RPMI/10% FBS.

### Arginase and Nitrite Assays

Arginase activity in cell lysates was measured based on the conversion of L-arginine to L-ornithine and urea according to the technique described by Corraliza and collaborators [38] with minor modifications. Briefly, cells were lysed for 30 min with 40 µL of 0,1% Triton-X-100. Thirty microliters of 25 mM Tris-HCl, pH 7.4 and 10 µL of 10 mM MnCl_2_ were added and the enzyme was heat-activated for 10 min at 56°C. Similar amounts of samples (40 µL) and 0.5 M L-arginine (pH 9.7) were mixed and incubated for 1 h at 37°C. The reaction was stopped by adding 400 µL of H_2_SO_4_ (96%), H_3_PO_4_ (85%), H_2_O (1/3/7, v/v/v). The urea concentration was measured at 540 nm after the addition of 8 µL of α-isonitropropiophenone 6%, followed by heating at 95°C for 30 min. Values were compared with a standard curve of urea concentration.

Nitrite concentrations were measured using the Greiss reaction [39]. In brief, 200 µL of the tested cell medium were incubated with 100 µL of 1% sulfanilamide and 100 µL of 0.3% N-1-naphthylethylenediamine dihydrochloride at room temperature for 5 min. Nitrite was quantified by spectrophotometry at 540 nm using sodium nitrite as standard.

### Determination of cytokine release

Cell medium was collected and TNF-α and IL-10 levels were determined by enzyme-linked immunoabsorbent assay (ELISA), according to the manufacturer's instructions (R&D Systems).

### Ectonucleotidase assay

ATPase, ADPase and AMPase activities were evaluated in 48-well plates containing macrophages that were washed three times with incubation medium in absence of nucleotides. The enzymatic reaction was started by the addition of 200 µL of incubation medium containing 2 mM CaCl_2_ (2 mM MgCl_2_ for AMPase assay), 120 mM NaCl, 5 mM KCl, 10 mM glucose, 20 mM Hepes, pH 7.4 and 2 mM ATP, ADP or AMP as substrates, at 37°C. After an incubation of 10 min, the reaction was stopped by transferring an aliquot of the incubation medium to a pre-chilled tube containing trichloroacetic acid (final concentration 5% w/v). The release of inorganic phosphate (P_i_) was measured by the malachite green method [40], using KH_2_PO_4_ as a P_i_ standard. Controls to determine nonenzymatic P_i_ release were performed by incubating the cells in the absence of the substrate, or the substrate in the absence of the cells that were added after the reaction had been stopped. All samples were run in triplicate. The protein concentration was measured by the Coomassie Blue method using bovine serum albumin as standard [41]. Specific activity was expressed as nmol P_i_ released/min/mg of protein.

### Analysis of extracellular ATP and AMP metabolism by HPLC

The cells were incubated as described above, except that ATP or AMP concentrations were 100 µM. To stop the reaction, an aliquot of the incubation medium was transferred to a pre-chilled tube and centrifuged at 4°C for 30 min at 16,000 *g*. Aliquots of 40 µL were applied to a reverse phase HPLC system using a C18 Shimadzu column (Shimadzu, Japan) with absorbance measured at 260 nm. The mobile phase was 60 mM KH_2_PO_4_, 5 mM tetrabutylammonium chloride, pH 6.0, in 30% methanol, as described [42]. Retention times were assessed using standard samples of ATP and its metabolites. The non-enzymatic hydrolysis of the ATP and AMP were consistently less than 5% of degradation. Cells incubated in incubation nucleotide free medium did not present any detectable peak.

### RT-PCR and qPCR

The RNA was isolated using the TRIzol Reagent (Invitrogen, Carlsbad, CA, USA). One µg of total RNA were added to each cDNA synthesis reaction using the SuperScript-III RT pre-amplification system (Invitrogen, Carlsbad, CA, USA). The RT-PCR reactions were performed in 25 µL of the reaction mixture containing 1 µL cDNA, 10 pmol of the primer in the supplied PCR mix buffer (Platinum PCR Supermix, Invitrogen). After initial denaturation for 5 min at 95°C, the amplifications were carried out for 35 cycles of denaturation at 94°C for 45 s, annealing at primer specific temperature for 45 s and extension at 72°C for 45 s. The PCR was ended by a7-min incubation at 72°C. The same program was used for the amplification of the reference gene β-actin. Sequences of primers are listed in supplementary Table S1. The PCR products were separated by 1.5% agarose gel electrophoresis and visualized with SyberGold (Molecular Probes).

qPCR was carried out in the Applied-Biosystem Step One Plus cycler using Platinum® SYBR® Green qPCR SuperMix-UDG (Invitrogen, Carlsbad, CA, USA) following the manufacturer's instructions and performed in triplicate. Total RNA and cDNA were generated as described above. The selected primers used for qPCR were the same used in RT-PCR analysis. All results were analyzed by the 2^−Δ/ΔCT^ method [43]. The β-actin was used as the internal control gene for all relative expression calculations.

### Flow Cytometry

The expression of selected surface proteins by peritoneal macrophages was evaluated by cytometry using the following antibodies (Ab): guinea pig polyclonal anti-mouse NTPDase1/CD39 (mN1-1_c_) [44], rabbit polyclonal anti-mouse NTPDase2 (mN2-36_l_) [45], guinea pig anti-mouse NTPDase3 (mN3-1_c_, I_4_) [44], rabbit anti-rat ecto-5′-nucleotidase/CD73 (rNu-9_l_) [46]–[47], anti-CD11b PE (BD Phamingen). The normal guinea pig and rabbit serum were used at a concentration commensurate to the experimental antibodies stains (polyclonals CD73, NTPDase1,2,3), 20–40 µg/mL. Briefly, before staining, the macrophages Fc receptors were blocked by incubating with Fc receptor blocking solution (Fc block: CD16/32, clone 2.4G2 from ATCC - HB-197) for 30 min in ice. After, the cells were incubated for 30 min with the above primary Ab diluted in PBS, 1% FBS, 0.1% sodium azide (PFA), and, when necessary, with secondary FITC-conjugated goat anti-rabbit IgG Ab (Invitrogen) or Alexa 488-conjugated goat anti-guinea pig IgG Ab (Invitrogen) for 30 min, with a minimum of two washes with PFA after each incubation. Cell surface fluorescence was measured with FACSCalibur Flow Cytometer (BD Biosciences).

### ATP-induced cell death assays

The cell death of peritoneal macrophages treated with ATP was determined based on two techniques: Trypan blue (0.1%) incorporation and propidium iodide (PI) staining (5 µM). In both assays, the cells were incubated with 2 mM ATP, with or without apyrase, or selective P2X7 antagonists for 3 h in an incubator with 5% CO_2_ atmosphere at 37°C. For the Trypan blue incorporation assay, the cell medium was removed after stimulation with ATP and cells were incubated with Trypan blue for 5 min, then washed twice with PBS and analyzed on microscope. For the PI staining assay, 5 µM PI was added in the culture medium 30 min before the end of ATP treatment, and after this time the medium was replaced to a new medium and cells were observed in an inverted microscope using a standard rhodamine filter set. Images were captured and then analyzed.

### Statistical analysis

Data were expressed as mean ± S.D. and were subjected to one-way analysis of variance (ANOVA) followed by Tukey's post-hoc test (for multiple comparisons). Differences between mean values were considered significant when P<0.05.

## Results

### Characterization of activated macrophages

**Figure 1:**
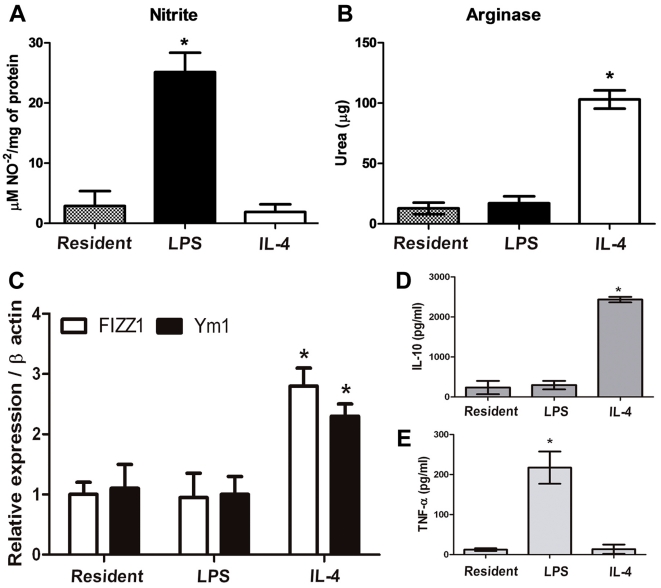
Characterization of resident, LPS- and IL-4-stimulated macrophages. (**A**) iNOS and (B) Arginase activities: iNOS activity was estimated by the NO^2−^ (nitrite) accumulation in the supernatant of cultured cells and Arginase activity was evaluated by measuring the formation of urea from arginine (3.10^5^ cells). *Significantly different from the two other groups (p<0.001). (**C**) FIZZ1 and Ym1 expression in stimulated macrophages was evaluated by qPCR. Expression was normalized to β-actin signals as described in Material and Methods. *Significantly different from resident and LPS-stimulated macrophages (p<0.001). (**D**) IL-10 and (**E**) TNF-α cytokines were measured from supernatants of macrophage cultures. *Significantly different from stimulated and resident macrophages (p<0.001). Data show mean ± SD of at least three independent experiments. Significant difference between groups was determined by ANOVA, followed by Tukey's test.

Primary macrophages were stimulated with LPS or IL-4 in order to obtain differentiated macrophages with distinct phenotypes, which were characterized by evaluating the arginase/iNOS activities, Ym1 and FIZZ1 mRNA expression and cytokine production. iNOS is up-regulated in response to inflammatory stimuli as macrophages shift towards the classical/M1 phenotype and become involved in the initiation of the immune response, while the expression of arginase is induced by Th2-type cytokines characterizing the alternative/M2 phenotype [2]–[3]. Figure 1A shows that, as expected, cells stimulated in culture with LPS exhibited an increase in the iNOS activity when compared to resident and IL-4 treated macrophages. In contrast, IL-4 treatment induced an increase in arginase activity (Fig. 1B).

To evaluate the spectrum of macrophage polarization, genes markers of macrophage differentiation were tested. It has been shown that the expression of Ym1 and FIZZ1 is strongly induced in alternative/M2 activated macrophages *in vivo* and *in vitro* in murine experimental models [3]. Accordingly, macrophages stimulated with IL-4 exhibited a strong induction of FIZZ1 and Ym1 expression when compared to resident and LPS-induced macrophages (Fig. 1C). Macrophage activation was also characterized by the production of inflammatory cytokines. The results showed that LPS-stimulation induced an increase of TNF-α release when compared to resident and IL-4 treated macrophages. In contrast, macrophages stimulated with IL-4 produced higher levels of IL-10 than resident and LPS-treated macrophages (Fig. 1D and E). Taken together, these results confirm that macrophages used in these experiments were differentiated into two phenotypes: macrophages classically activated by LPS (M1) and alternatively activated by IL-4 (M2), and this protocol was applied to further experiments as described below.

### Characterization of the expression of purinergic receptors and ectonucleotidases in M1 and M2 macrophages

Since extracellular nucleotides are associated with immune/inflammatory responses through purinergic receptor activation, the presence of P1 and P2 mRNAs in differentiated macrophages was analyzed by RT-PCR. As shown in Table 1, similar purinergic receptor expression in M1 and M2 activated-macrophages was observed. Considering that the availability of extracellular adenine nucleotides is controlled by the concerted action of NTPDases and ecto-5′-NT, the qualitative analysis of resident, M1 or M2 stimulated macrophages showed the presence of NTPDase1, -2, -3 and ecto-5′-NT mRNA transcripts, while NTPDase8 was absent (Table 1).

**Table 1:** Expression of P1 and P2 receptors, and ectonucleotidases, as determined by RT-PCR.

Genes	Resident	M1	M2
***P2rx1***	−	−	−
***P2rx2***	−	−	−
***P2rx3***	−	−	−
***P2rx4***	+	+	+
***P2rx5***	−	−	−
***P2rx6***	−	−	−
***P2rx7***	+	+	+
***P2ry1***	+	+	+
***P2ry2***	+	+	+
***P2ry4***	−	−	−
***P2ry6***	±	±	±
***P2ry12***	±	±	±
***P2ry13***	−	−	−
***P2ry14***	+	+	+
***Adora1 (A1)***	±	±	±
***Adora2a (A2A)***	+	+	+
***Adora2b (A2B)***	±	±	±
***Adora3 (A3)***	+	+	+
***Entpd1***	+	+	+
***Entpd2***	±	±	±
***Entpd3***	±	±	±
***Entpd8***	−	−	−
***Nt5e***	+	+	+

The table shows gene/β-actin ratio determined after electrophoresis on a 1.5% agarose gel stained with SyberGold and visualization under ultraviolet light by using ImageJ 1.37 for Windows. Low DNA Mass Ladder (Invitrogen) was used as a molecular marker. The genes with gene/β-actin ratio ≥0.7 were considered strong expression (+), genes with ratio ≤0.3 were considered as barely detectable (±) while genes without any signal detected were considered as negative (−).

### Ectonucleotidase activity in M1 and M2 macrophages

**Figure 2:**
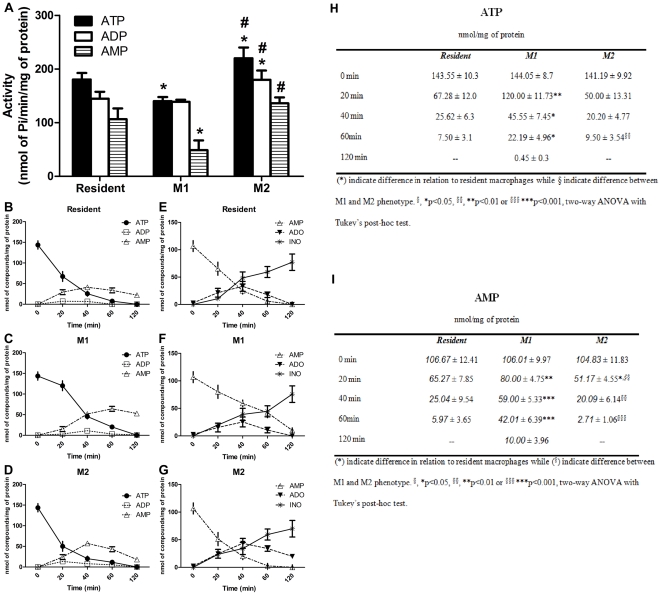
NTPDase activity on mouse macrophages after phenotype differentiation. (**A**) Resident, M1 (stimulated with LPS) and M2 (stimulated with IL-4) macrophages were incubated in 48-well plates with ATP, ADP or AMP as described in Materials and Methods (section 2.5). Specific activity values were expressed as nmol Pi/min/mg protein. The data represent the mean ± S.D. (n = 5) with pooled macrophages from 6 to 8 mice *per* experiment carried out separately. Data were analyzed by ANOVA, followed by Tukey's test. (*) Significantly different from resident macrophages; (^#^) significantly different from LPS-stimulated macrophages (p<0.05). (**B–D**) Metabolism of extracellular ATP by HPLC; resident (B), M1 (stimulated with LPS) (C) and M2 (stimulated with IL-4) (D) macrophages were incubated in 48-well plates with 100 µM ATP in 200 µl of incubation medium as described in Material and Methods. An aliquot of the supernatant was withdrawn at 0, 20, 40, 60 and 120 min and the presence of ATP, ADP, AMP were determined. Data are mean ± SD values from three experiments in triplicates. (**E–G**) The same procedure utilized to evaluate the metabolism extracellular of ATP was used to AMP metabolism - adenosine (ADO) and inosine (INO) - Resident (E), M1 (stimulated with LPS) (F) and M2 (stimulated with IL-4) macrophages (G). Data are mean ± SD values from three experiments in triplicates. (**H**, **I**) Amount of nucleotides/nucleosides at different time of incubation. Data are mean ± SD values from three experiments in triplicates.

The differential capacity of resident, M1 or M2 macrophages to hydrolyze extracellular ATP, ADP and AMP was investigated. Macrophages M1 exhibited a decrease in the ATPase (20%) and AMPase (54%) activities when compared to resident macrophages, whereas the ADP hydrolysis was not altered (Fig. 2A). In contrast, macrophages M2 showed a significant increase in ATP (∼20%) and ADP (∼25%) hydrolysis, while no changes were observed in AMP hydrolysis when compared to resident macrophages. Noteworthy, ATP, ADP and AMP hydrolysis was significantly higher in M2 macrophages when compared to M1 cells, suggesting that in M2 macrophages the nucleotide pathway is directed through the generation of anti-inflammatory adenosine.

The analysis of the catabolism of nucleotides by HPLC showed that in M1 macrophages, ATP was slowly metabolized along the 2 h incubation, in comparison to resident macrophages, being converted to ADP and AMP (Fig. 2B, C). Confirming the higher ATPase activity in M2 macrophages, ATP was nearly completely converted to AMP in about 20 min (Fig. 2D). In addition, the evaluation of AMP hydrolysis indicated a lower AMPase activity in the M1 phenotype when compared to resident and M2 cells (Fig. 2E, F). The HPLC profile of AMP metabolism confirms the results of the figure 2A and shows a higher level of adenosine in the extracellular medium of M2 macrophages from 40 min to 120 min. The inosine levels progressively increased along the incubation time in all phenotypes (Fig. 2G).

### Differential ectonucleotidase expression in M1 and M2 macrophages

**Figure 3:**
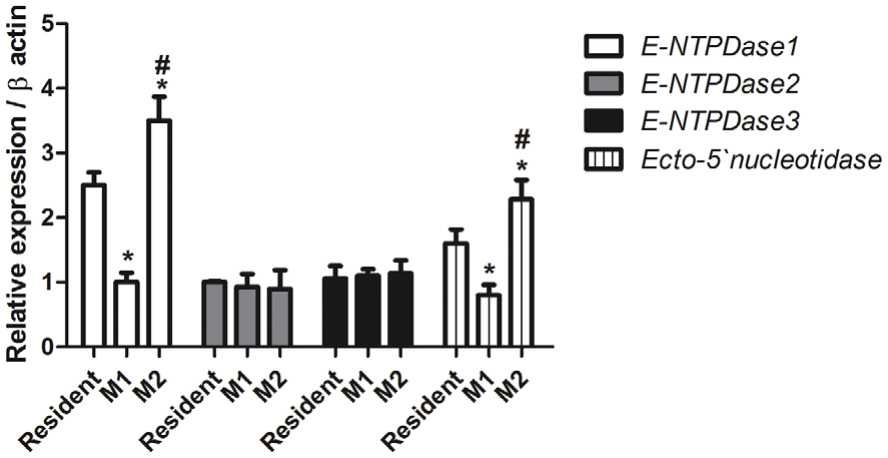
PCR quantification of NTPDase1, -2, -3 and ecto-5′-nucleotidase mRNAs. The total mRNA amount were normalized to β-actin signals and expressed as 2^−Δ/ΔCT^. Data show mean±SD for real time PCR experiments performed in triplicate with RNA purified from three independent experiments with pooled macrophages from 8 to 10 mice per experiment. M1 (stimulated with LPS) and M2 (stimulated with IL-4) macrophages were compared to resident macrophages (*) p<0.001, and (#) p<0.01 M2 (stimulated with IL-4) compared to M1 (stimulated with LPS) macrophages, two-way ANOVA with Tukey's post-hoc test.

**Figure 4:**
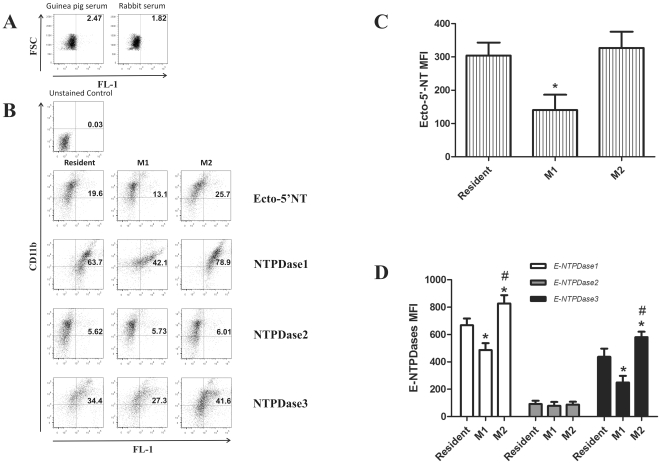
Activated macrophages express different protein levels of NTPDases and Ecto-5′-nucleotidase. The cells were stained 24 h after stimulation with antibodies to CD11b and to NTPDase1, -2 and -3, or Ecto-5′NT, and when necessary, with secondary FITC- or Alexa 488-conjugated antibodies. Macrophages were primed either with LPS to generate M1 macrophages, with IL-4 to generate M2 macrophages, or left unstimulated (resident). FL-1 represent the intensity of staining with the indicated Abs. (**A**) The controls were performed using guinea pig serum (Sigma) and rabbit serum (Invitrogen) as primary antibodies as detailed in Materials and Methods section. The results presented were subtracted from the data presented in the following panels according to the Abs used. (**B**) Dot plot with percentage of double positive cells for CD11b and NTPDase1, -2, -3 or Ecto-5′NT. (**C**) Mean fluorescence intensity (MFI) from panel B is shown for Ecto-5′NT and (**D**) depicts NTPDase1, -2, -3 MFIs. Data are representative of at least three independent experiments with pooled macrophages from 7 to 9 mice per experiment. (*) p<0.05 indicates changes in expression when compared against resident macrophages and (#) p<0.05 indicate significant difference between M1 and M2 macrophages, two-way ANOVA with Tukey's post-hoc test.

Considering the differential nucleotide metabolism pattern presented by resident, M1 and M2 macrophage phenotypes, a qPCR analysis of the mRNA levels of NTPDase1, -2, -3 and ecto-5′-NT was evaluated. A significant decreased of mRNA expression of NTPDase1 and ecto-5′-NT was observed in M1 macrophages when compared to resident cells. On the other hand, the mRNA expression of NTPDase1 and ecto-5′-NT in M2 macrophages was significantly increased in relation to resident macrophages and M1 cells. The mRNA of NTPDase2 and -3 did not differ among the phenotypes (Fig. 3). In addition, the protein levels were evaluated by flow cytometry using specific antibodies. A decrease in protein expression of NTPDase1, -3 and ecto-5′-NT was detected in the M1 macrophages in relation to resident macrophages (Fig. 4A, B, C). In contrast, M2 macrophages exhibited an increase of NTPDase1 and -3 expression in relation to resident and M1 macrophages, while the ecto-5′-NT protein expression was not altered in comparison to resident cells (Fig. 4A, B, C). The NTPDase2 did not present any change at protein level during differentiation of macrophages (Fig. 4A). Thus, the three macrophage populations express distinct levels of these molecules, which may reveal distinct functions related to the modulation of purinergic receptor activation during the ongoing inflammatory process.

### M1 macrophages are more susceptible to ATP-induced cell death

**Figure 5:**
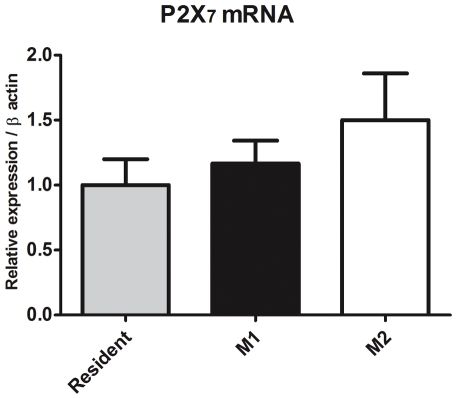
PCR quantification of P2X7 mRNA. The total mRNA amount were normalized to β-actin signals and expressed as 2^−Δ/ΔCT^. Data show mean±SD for real time PCR experiments performed in triplicate with RNA purified from three independent experiments with pooled macrophages from 5 mice per experiment. There were no significant differences in the P2X7 mRNA among the groups.

**Figure 6:**
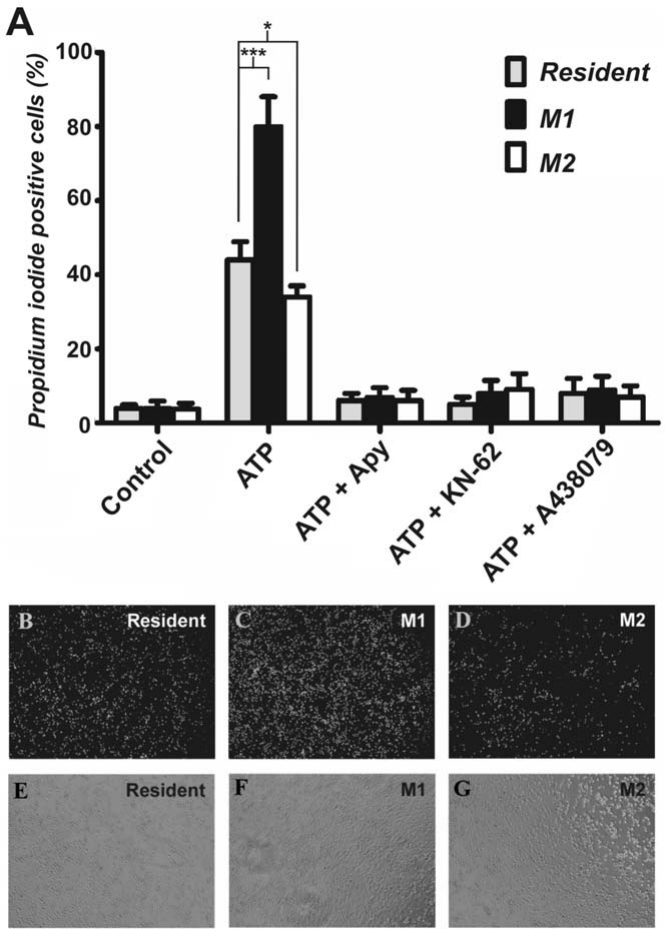
The NTPDase activity on macrophages after phenotype differentiation alters the susceptibility to ATP induced cell death. (**A**) After macrophages differentiation these cells were treated for 3 h with 2 mM ATP in the presence or absence of potato apyrase (apy; 2 U) or P2X7 receptor antagonists (3 µM KN-62, 10 µM A438079). The total number of the cells was counted in five random fields in visible filter and the cells positive for PI were counted in same fields but with ultra-violet filter. Data show mean±SD of three experiments. Significantly different from resident macrophages for ***p<0.001 or *p<0.05, two-way ANOVA with Tukey's post-hoc test. (**B**, **C**, **D**) UV and (**E**, **F**, **G**) visible representative images (magnification: 40×). M1 (stimulated with LPS) and M2 (stimulated with IL-4) macrophages were compared to resident macrophages.

To assess whether NTPDases can regulate P2 function during macrophage differentiation, we evaluated the P2X7-associated function in cell death [48]–[49]. Firstly, we verified that the mRNA expression of P2X7 did not significantly differ during macrophage activation (Fig. 5). Next, we compared the effects of ATP on the mortality of M1 and M2 macrophages. Quantification of PI fluorescence showed that 2 mM ATP caused ∼44% cell death in resident, 80% in M1 and 34% in M2 macrophages (Fig. 6A–G). Similar data were obtained for Trypan blue incorporation (data not shown). Accordingly, 2 U apyrase, an ATP scavenger, prevented the cell death induced by ATP in all macrophage phenotypes. In addition, and in accordance to the role of P2X7 in ATP-induced macrophage death, two selective P2X7 receptor antagonists KN-62 and A438079 prevented this effect (Fig. 6A). Taken together, these results suggest that the modulation of NTPDase expression in macrophages observed during M1 and M2 differentiation could be important in the regulation of P2X7-induced cell death.

## Discussion

In the present study in order to characterize the participation of ectonucleotidases during macrophage differentiation, and also in the inflammatory processes, we differentiated peritoneal macrophages into two phenotypes *in vitro*: a pro-inflammatory phenotype obtained by the stimulation with LPS (named classical/M1), and an anti-inflammatory phenotype obtained by the stimulation with IL-4 (named alternative/M2). As expected, macrophages of M1 phenotype exhibited increased iNOS, high levels of the pro-inflammatory cytokine TNF-α and reduced arginase activities, while these characteristics were opposite in macrophages of the M2 phenoype. In addition, the anti-inflammatory cytokine IL-10 was higher in M2 than M1 macrophages and the high expression of genes FIZZ1 and Ym1 [50]–[51]. Taken together, the results presented in figure 1 ensure that macrophages were differentiated into two extreme phenotypes M1 and M2.

We demonstrated that NTPDase1, -2, -3 as well as ecto-5′-NT are expressed in resident, M1 (LPS) and M2 (IL-4) macrophages (Table 1). In a previous study, Lévesque and collaborators [52] have shown the presence of NTPDase1 and a modest expression of NTPDase2 in thioglycollate-elicited macrophages, but they failed to identify either the mRNA or the protein of NTPDase3, or of ecto-5′-NT. In agreement with these results in preliminary experiments, we did not detect any AMPase activity or NTPDase3 expression in thioglycollate-elicited macrophages (data not shown). Then, it is possible that thioglycollate alters the expression of ectonucleotidases. For this reason, in the present study, we have opted for using resident peritoneal macrophages from naïve mice. Noteworthy, Lévesque and collaborators [52] demonstrated weak ATPase and ADPase activities in *Entpd1^−/−^* in relation to *Entpd1^+/+^* macrophages, which revealed the dominant NTPDase1 activity in macrophages.

The results presented herein showed a reduction in the ATP hydrolysis (Fig. 2A and 2C) in M1 macrophages, which is probably due to decrease in the NTPDase1 protein expression (Fig. 4A). In addition to the post-transcriptional effect, previous studies demonstrated loss of NTPDase1 activity in endothelial cells after exposure to LPS [53], which can be explained by the possible alterations in the membrane structure due to the LPS-evoked inflammation. This explanation may also be plausible for other NTPDases, as these enzymes are sensitive to transmembrane changes [53]–[54]. Otherwise, there was no alteration in ADP hydrolysis in M1 macrophages in relation to resident cells. The possible explanation for this is that the marked decrease in the expression of NTPDase1 could highlight the humble NTPDase2 activity. In M2 macrophages, the results showed that ATPase and ADPase activities increased in relation to resident and M1 macrophages, which was accompanied by an increase in the NTPDase1 protein expression. Despite the observed alterations of NTPDase3 protein expression in the two phenotypes (M1 and M2), the levels mRNA were unaltered, what could be explained by a feedback auto-regulatory loop at transcriptional level [55]. In summary, the M1 macrophages may accumulate ATP, which might in turn favor ATP-mediated pathogen clearance and P2 activation. In addition to the observed alterations in ATP and ADP hydrolysis, the present study demonstrated that M1 macrophages displayed a decrease of ecto-5′-NT activity in relation to resident macrophages, which was accompanied by a reduction in the expression of mRNA (Fig. 3) and protein levels (Fig. 4B). A probable explanation for the decreased ecto-5′-NT activity in macrophages stimulated by LPS (M1) is the activation of the transcription factor NF-κB, which may suppress the transcription of the gene encoding ecto-5′-NT [56]. This notion is supported by studies that have demonstrated that anti-inflammatory effects of methotrexate results from the activation of ecto-5′-NT through suppression of NF-κB [57].

Recently, it was described that macrophages are able to dynamically switch from a proinflammatory to an anti-inflammatory state [58]. Here, we show that M2 macrophages present increased ATPase, ADPase and ecto-5′-NT activities in relation to M1 phenotype, what can drive the ATP rapidly to generation of adenosine in this phenotype. The extracellular adenosine is a metabolite produced, mainly by breakdown of ATP, which is elevated during the inflammation process and is associated with anti-inflammatory and regeneration actions in immune cells [22]. Another interesting result observed on M2 macrophages was the production of adenosine and inosine after AMP hydrolysis (Fig. 2G). Although inosine has been suggested to participate in inflammatory process, there is a lack of evidence about this [59]–[60]. In contrast, the role of adenosine and its receptors in the control of immune response, including macrophage activation, is well established [17], [22]. In this scenario, we can suggest that production of adenosine would be more relevant to the anti-inflammatory and regeneration actions of M2 macrophage phenotype. Therefore, the NTPDases and ecto-5′NT combined activities could rapidly convert ATP to adenosine in order to impair P2 receptor stimulation and, as a consequence, facilitate P1 receptor stimulation.

Finally, in this study, we showed that mRNA P2X7 expression was not altered during differentiation (Fig. 5), which are in accordance with data from Pelegrin and collaborators [61] that showed similar P2X7 protein expression along macrophage differentiation (M1 to M2). In addition, we provide novel evidence indicating that macrophages of the M1 phenotype are more susceptible to cell death via activation of P2X7, in comparison to M2 macrophages, which have a higher ATPase activity (Fig. 6A–G). These results are in agreement with previous studies that report the importance of NTPDases in the P2X7 functionality [52], [62].

In conclusion, the compelling evidence provided by us indicates that changes in the expression of NTPDases and ecto-5′-NT in macrophages during phenotypic differentiation (M1 to M2) triggers a progressive decrease in nucleotide concentrations and an increase in adenosine availability. Therefore, these changes in ectoenzyme activities might allow macrophages to adjust the outcome of the extracellular purinergic cascade in order to fine-tune their functions during the inflammatory set.

## Supporting Information

Table S1 **PCR primers for purinergic receptors and ectonucleotidases.**
(DOC)
