# Supplementary material for: Differential Macrophage Activation Alters the Expression Profile of NTPDase and Ecto-5′-Nucleotidase
Source: PLoS One. 2012 Feb 13;7(2):e31205. doi: 10.1371/journal.pone.0031205 (PMC3278434; doi:10.1371/journal.pone.0031205)
Supplement: Table S1 — PCR primers for purinergic receptors and ectonucleotidases. (DOC) [file pone.0031205.s001.doc]

**TABLE S1.**

| Primer | Sense | Antisense |
| --- | --- | --- |
| ***P2rx1*** | 5`-AAGGTCAACAGGCGCAACC-3` | 5`-AACACCTTGAAGAGGTGACG-3` |
| ***P2rx2*** | 5`-GTGCAGAAAAGCTACCAGG-3` | 5`-GGATGGTGAAATTTGGGGC-3` |
| ***P2rx3*** | 5`-GCTGCGTGAACTACAGCTC-3` | 5`-ACTGGTCCCAGGCCTTGTC-3` |
| ***P2rx4*** | 5`-CTTGGATTCCGGATCTGGG-3` | 5`-GGAATATGGGGCAGAAGGG-3` |
| ***P2rx5*** | 5`-GCACCTGTGAGATCTTTGC-3` | 5`-TCGGAAGATGGGGCAGTAG-3` |
| ***P2rx6*** | 5`-CAGGACCTGTGAGATCTGG-3` | 5`-TCCTGCAGCTGGAAGGAGT-3` |
| ***P2rx7*** | 5`-TCCCTTTGCAGGGGAACTC-3` | 5`-GTACGGTGAAGTTTTCGGC-3` |
| ***P2ry1*** | 5`-TGTTCAATTTGGCTCTGGC-3` | 5`-AGATGAAATAACTTCGCAGG-3` |
| ***P2ry2*** | 5`-CTTCGCCCTCTGCTTCCTG-3` | 5`-TTGGCATCTCGGGCAAAGC-3` |
| ***P2ry4*** | 5`-GGCATTGTCAGACACCTTG-3` | 5`-AAGACAGTCAGCACCACAG-3` |
| ***P2ry6*** | 5`-CGCTTCCTCTTCTATGCCA-3` | 5`-AGGCTGTCTTGGTGATGTG-3` |
| ***P2ry12*** | 5`-GACTACAAGATCACCCAGG-3` | 5`-CCTCCTGTTGGTGAGAATC-3` |
| ***P2ry13*** | 5`-GCCGACTTGATAATGACAC-3` | 5`-ATGATCTTGAGGAATCTGTC-3` |
| ***P2ry14*** | 5`-TCTTTTACGTGCCCAGCTC-3` | 5`-CTGTCAAAGCTGATGAGCC-3` |
| ***Entpd1*** | 5`-AGCTGCCCCTTATGGAAGAT-3` | 5`-TCAGTCCCACAGCAATCAAA-3` |
| ***Entpd2*** | 5`-TTCCTGGGATGTCAGGTCTC-3 | 5`-GTCTCTGGTGCTTGCCTTTC-3` |
| ***Entpd3*** | 5`-ACCTGTCCCGTGCTTAAATG`-3` | 5`-AGACAGAGTGAAGCCCCTGA-3` |
| ***Entpd8*** | 5`-CACACAGGACCTTCTGAGCA-3` | 5`-AGCCTTCTGAGGTGGCACTA-3` |
| ***Nt5e*** | 5`-CAGGAAATCCACCTTCCAAA-3` | 5`-AACCTTCAGGTAGCCCAGGT-3` |
| ***Retnla*** | 5`-TCCCAGTGAATACTGATGAGA-3` | 5`-CACTCTGGATCTCCCAAGA-3` |
| ***Chi3l3*** | 5`-GGGCATACCTTTATCCTGAG-3` | 5`-CCACTGAAGTCATCCATGTC- 3` |
| ***β-actin*** | 5`- TATGCCAACACAGTGCTGTCTGG-3` | 5`-TACTCCTGCTTGCTGATCCACAT-3` |
